# Real-time RT-PCR high-resolution melting curve analysis and multiplex RT-PCR to detect and differentiate grapevine leafroll-associated virus 3 variant groups I, II, III and VI

**DOI:** 10.1186/1743-422X-9-219

**Published:** 2012-09-27

**Authors:** Rachelle Bester, Anna E C Jooste, Hans J Maree, Johan T Burger

**Affiliations:** 1 Department of Genetics, Stellenbosch University, Stellenbosch, South Africa; 2 Plant Protection Research Institute, Agricultural Research Council, Pretoria, South Africa; 3 Biotechnology Platform, Agricultural Research Council Infruitec-Nietvoorbij, Stellenbosch, South Africa

**Keywords:** Grapevine leafroll-associated virus 3, GLRaV-3, Real-time RT-PCR, High-resolution melting curve analysis, Multiplex RT-PCR, Molecular variants, Leafroll disease

## Abstract

**Background:**

Grapevine leafroll-associated virus 3 (GLRaV-3) is the main contributing agent of leafroll disease worldwide. Four of the six GLRaV-3 variant groups known have been found in South Africa, but their individual contribution to leafroll disease is unknown. In order to study the pathogenesis of leafroll disease, a sensitive and accurate diagnostic assay is required that can detect different variant groups of GLRaV-3.

**Methods:**

In this study, a one-step real-time RT-PCR, followed by high-resolution melting (HRM) curve analysis for the simultaneous detection and identification of GLRaV-3 variants of groups I, II, III and VI, was developed. A melting point confidence interval for each variant group was calculated to include at least 90% of all melting points observed. A multiplex RT-PCR protocol was developed to these four variant groups in order to assess the efficacy of the real-time RT-PCR HRM assay.

**Results:**

A universal primer set for GLRaV-3 targeting the heat shock protein 70 homologue (Hsp70h) gene of GLRaV-3 was designed that is able to detect GLRaV-3 variant groups I, II, III and VI and differentiate between them with high-resolution melting curve analysis. The real-time RT-PCR HRM and the multiplex RT-PCR were optimized using 121 GLRaV-3 positive samples. Due to a considerable variation in melting profile observed within each GLRaV-3 group, a confidence interval of above 90% was calculated for each variant group, based on the range and distribution of melting points. The intervals of groups I and II could not be distinguished and a 95% joint confidence interval was calculated for simultaneous detection of group I and II variants. An additional primer pair targeting GLRaV-3 ORF1a was developed that can be used in a subsequent real-time RT-PCR HRM to differentiate between variants of groups I and II. Additionally, the multiplex RT-PCR successfully validated 94.64% of the infections detected with the real-time RT-PCR HRM.

**Conclusion:**

The real-time RT-PCR HRM provides a sensitive, automated and rapid tool to detect and differentiate different variant groups in order to study the epidemiology of leafroll disease.

## Background

Grapevine leafroll-associated virus 3 (GLRaV-3) is a positive-sense single-stranded RNA virus that is the type member of the genus *Ampelovirus* in the family *Closteroviridae*[1]. This virus is phloem-limited and is considered the main contributing agent of leafroll disease worldwide with detrimental effects on both wine and table grapes. Six variant groups of GLRaV-3 have been identified of which four are known to be present in South Africa [2-8]. The genomes of at least one representative isolate of variant groups I, II, III and VI have been sequenced. These are isolates 621, WA-MR, NY-1 and CI-766 (group I) [2-5], 623 and GP18 (group II) [2,6], and PL-20 (group III) [2]. Recently, isolates GH11 and GH30 (group VI), were identified, and showed less than 70% nucleotide identity to other GLRaV-3 variant groups [7]. Limited sequence information for GLRaV-3 variant groups IV and V is available and isolates from these groups are only represented by coat protein gene sequences in the GenBank database [8]. All these genetic variants commonly occur as mixed infections. However, no specific disease symptoms or geographic distribution could so far be assigned to a specific variant group or cluster of variant groups. It is therefore necessary to develop an effective method that can detect all GLRaV-3 variants and differentiate between them. Previously, single-strand conformation polymorphism (SSCP) profiles have been used to investigate the population structure and genetic variability of GLRaV-3 variants [2,9]. Although SSCP analysis is fast and cost effective for variant typing based on sequence heterogeneity, the technique is not as sensitive as RT-PCR and requires sequencing to verify new variants. Metagenomic sequencing or next generation sequencing is the most sensitive diagnostic tool available to detect and identify known and novel viruses [10-13]. Next generation sequencing can identify viral pathogens occurring at extremely low titers without the necessity of any prior sequence knowledge. Although this technique is unbiased, it is still too expensive to use for routine diagnostics. Reverse transcription polymerase chain reaction (RT-PCR) is a diagnostic tool capable of detecting virus sequences at low concentrations and can be designed to be genus-, species-, or isolate-specific [14,15]. The design of optimal RT-PCR primers requires accurate sequence information. The recently sequenced GLRaV-3 group VI was found to be less than 70% similar to other GLRaV-3 variant groups and warrants a re-evaluation of existing GLRaV-3 RT-PCR diagnostic primers.

Real-time RT-PCR is another technique that has been successfully utilized to detect various plant viruses, including GLRaV-3 [16]. It is a rapid, reliable and quantitative detection method that is more sensitive than conventional RT-PCR. It has the potential for multiplexing and is therefore able to detect several pathogens in the same reaction [16]. The development of high-resolution melting (HRM) curve analysis, as an extension to real-time RT-PCR, provides a rapid, high-throughput, cost effective and single tube approach to discriminate and genotype strains of bacteria and viruses [17]. The genotyping of variants does not require a labeled probe and sequence variants can be distinguished from each other based on their individual melting temperatures [18]. High-resolution melting (HRM) curve analysis was effectively applied in diagnostics for viruses affecting humans [19] as well as for phytopathogenic bacteria [17].

The aim of this study was to develop a simple and reliable one-step real-time RT-PCR assay with high-resolution melting (HRM) curve analysis (RT-PCR HRM) for the simultaneous detection and identification of GLRaV-3 variants of groups I, II, III and VI, all four previously detected in South African vineyards. To achieve this, a universal primer set, able to detect and differentiate these variant groups, was designed. A multiplex RT-PCR was also developed to validate the RT-PCR HRM. The application of these protocols will aid in the understanding of the molecular epidemiology of GLRaV-3 and leafroll disease and assist programmes focused at managing and controlling the spread of GLRaV-3.

## Results and discussion

### Primer design and evaluation

**Figure 1:**
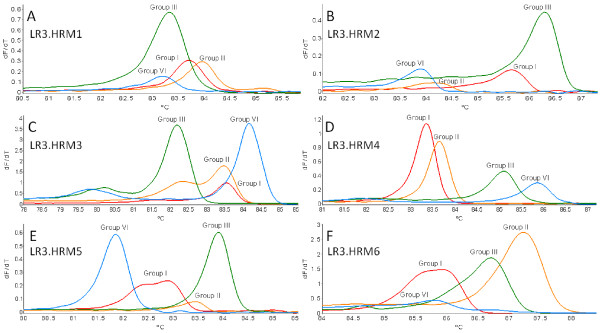
**Comparison of primer pairs evaluated for their ability to detect and differentiate between GLRaV-3 variant groups.** Derivative HRM curves (dF/dT) obtained using RNA extracted from plants singly infected with only one variant group of GLRaV-3 in the real-time RT-PCR HRM assay.

**Figure 2:**
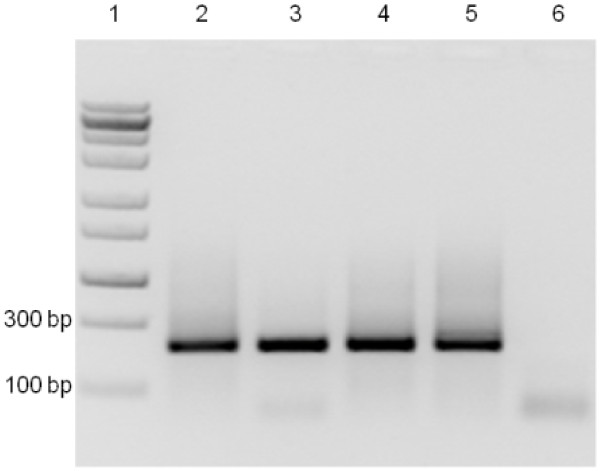
**Agarose gel electrophoresis of RT-PCR HRM amplicons.** Visualization of amplicons generated with the real-time RT-PCR HRM assay with primer pair LR3.HRM4, separated on a 1.5% TAE agarose gel with ethidium bromide staining. Lane 1: Fermentas Zipruler Express DNA ladder 2, Lane 2: Group I, Lane 3: Group II, Lane 4: Group III, Lane 5: Group VI, Lane 6: RNA negative control.

Six primer pairs were evaluated for their ability to detect and differentiate between GLRaV-3 variant groups I, II, III and VI, utilizing the RT-PCR HRM (Figure 1). From the six primer pairs evaluated for the RT-PCR HRM, primer pairs LR3.HRM1 and LR3.HRM3 were eliminated since they were unable to differentiate between groups III and VI, and groups I and II, respectively (Figure 1A and Figure 1C). The amplification efficiency for group II variants by primer pairs LR3.HRM2 and LR3.HRM5 were sub-optimal (Figure 1B and Figure 1E). Only primer pairs LR3.HRM4 (Figure 1D) and LR3.HRM6 (Figure 1F) showed reproducible results, with primer pair LR3.HRM4 yielding equal amplification for all variant groups, based on electrophoretic analysis. Primer pair LR3.HRM4 (Figure 1D) produced a single PCR product of 226 bp for each variant group when visualized on a 1.5% TAE agarose gel (Figure 2). After HRM curve analysis the LR3.HRM4 primer pair produced one melting peak each for group I and II variants with average melting points of 83.60°C and 83.77°C, respectively (Table 1). Variant groups III and VI both produced a major peak together with a smaller shoulder peak. The average melting points of the major melting peak for groups III and VI were 85.44°C and 85.97°C, respectively (Table 1). The shoulder peaks produced by the LR3.HRM4 primer pair for groups III and VI was not regarded as unspecific amplification since only one band was detected after gel electrophoresis. Therefore, it is likely that the shoulder peaks can be the result of uneven G/C distribution throughout the targeted RNA of groups III and VI [20,21]. The last 126 nucleotides at the 3’ end of the 226 bp amplicon for groups III and VI have an average GC content of 53% and 56% respectively, compared to the 47.5% and 46% for groups I and II.

**Table 1:** Descriptive statistics of melting points generated by real-time RT-PCR HRM assays with primer pairs LR3.HRM4 and LR3.HRM6

**Variant group**	**Number of data points**^**a**^	**Min**	**Max**	**Mean**	**Temperature range between upper and lower limit**
**LR3.HRM4**					
Group I	31	83.20	83.98	83.60	0.78
Group II	203	83.15	84.25	83.77	1.10
Group III	73	84.87	85.70	85.44	0.83
Group VI	142	85.30	86.37	85.97	1.07
**LR3.HRM6**					
Group I	27	84.78	85.42	85.03	0.64
Group II	187	85.95	86.90	86.41	0.95

^a^Number of melting point temperatures generated per variant group from the 121 samples. More than one melting point temperature per sample was generated due to mix infections and duplex reactions.

No discriminatory difference could be detected between the average melting points of groups I and II (Table 1). Pairwise nucleotide sequence comparisons showed that there are only up to 11 nucleotide differences between GLRaV-3 group I and group II within the targeted region, whereas the other variant groups had 24-61 nucleotide differences (Table 2). Although primer pair LR3.HRM6 was unable to detect group VI variants, it could efficiently differentiate between group I and II variants (Figure 1F). It produced a single melting peak on a derivative melting curve for both variant groups I and II with average melting points of 85.03°C and 86.41°C, respectively (Table 1). Sequence analysis performed on the Hsp70h gene sequences available on GenBank, spanning the LR3.HRM4 primer pair target region, indicates that the LR3.HRM4 primer pair will be able to detect all variants from groups I, II, III and VI. Unfortunately groups IV and V could not be included in this study as only coat protein sequences of these variant groups are available.

**Table 2:** Pairwise comparison of LR3.HRM4 amplicon (226 nt segment of Hsp70h) for each variant group

		**Variant group representative isolates**									
		**1**	**2**	**3**	**4**	**5**	**6**	**7**	**8**	**9**	**10**
Group I_GU983863.1_GLRaV-3_Isolate_WA-MR	1		**0**	**1**	**1**	**9**	**10**	**24**	**50**	**50**	**53**
Group I_AF037268.2_GLRaV-3_Isolate_NY-1	2	100		**1**	**1**	**9**	**10**	**24**	**50**	**50**	**53**
Group I_EU344893.1_GLRaV-3_Isolate_Cl-766	3	99.56	99.56		**2**	**9**	**10**	**24**	**50**	**50**	**53**
Group I_GQ352631.1_GLRaV-3_Isolate_621	4	99.56	99.56	99.12		**10**	**11**	**25**	**49**	**49**	**52**
Group II_GQ352632.1_GLRaV-3_Isolate_623	5	96.02	96.02	96.02	95.58		**2**	**27**	**49**	**49**	**53**
Group II_EU259806.1_GLRaV-3_Isolate_GP18	6	95.58	95.58	95.58	95.13	99.12		**27**	**51**	**51**	**53**
Group III_GQ352633.1_GLRaV-3_Isolate PL-20	7	89.38	89.38	89.38	88.94	88.05	88.05		**58**	**58**	**61**
Group VI_JQ655295_GLRaV-3_Isolate GH11	8	77.88	77.88	77.88	78.32	78.32	77.43	74.34		**0**	**10**
Group VI_JQ655296_GLRaV-3_Isolate_GH30	9	77.88	77.88	77.88	78.32	78.32	77.43	74.34	100		**10**
Group VI_EF508151.1_GLRaV-3_Isolate_NZ-1	10	76.55	76.55	76.55	76.99	76.55	76.55	73.01	95.58	95.58	

The upper comparison in bold is the number of nucleotide differences between variant sequences and the lower comparison is the percent identity (%) between variant sequences.

### Verification of one-step real-time RT-PCR HRM assay

**Figure 3:**
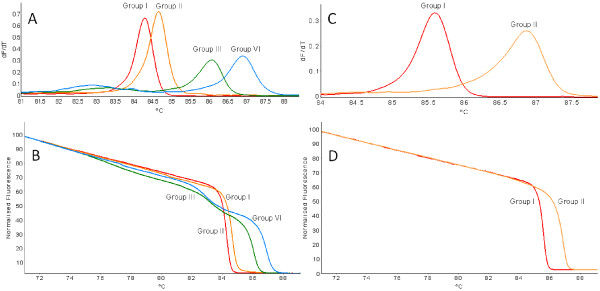
**High-resolution melting curve analysis using variant-specific plasmid DNA in real-time PCR HRM assays.** Derivative HRM curves (dF/dT) (**A** and **C**) and normalized HRM curves (**B** and **D**) obtained using SYTO 9 for the detection of GLRaV-3 variants. Primer pair LR3.HRM4 is represented by **A** and **B** and primer pair LR3.HRM6 is represented by **C** and **D**.

**Figure 4:**
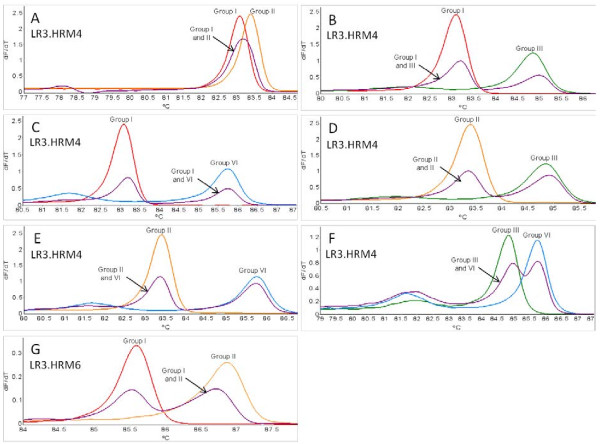
**Variant-specific plasmid DNA duplex infections.** Comparison of the different duplex infections possible between variant groups I, II, III and VI. Derivative HRM curves (dF/dT) obtained using primer pairs LR3.HRM4 (**A-F**) and LR3.HRM6 (**G**) in real-time PCR HRM assays using variant specific plasmid DNA. All mixed infections shown are the 1:1 duplex artificial mix compared to melting curves generated from single variant reactions.

Variant-specific plasmids containing the amplicons from primer pairs LR3.HRM4 and LR3.HRM6 were constructed. The derivative HRM curves (dF/dT) and normalized HRM curves generated by using the variant-specific plasmid DNA in the real-time PCR HRM assay (PCR HRM) (Figure 3) verified the melting curves observed when singly infected plant RNA samples were screened (Figure 1D and Figure 1F). Duplex artificially mixed infections between the variant-specific plasmid DNA confirmed that primer pair LR3.HRM4 can differentiate between mixed infections (Figure 4B-F), except for mixed infections of variants from groups I and II (Figure 4A). The duplex artificial mix with the variant-specific plasmid DNA of groups I and II illustrated that a single melting peak is produced on the derivative melting curve (Figure 4A). This melting peak was not distinguishable from either the singly infected group I or II melting peaks, based on the confidence intervals calculated. In order to classify a sample as group I and/or II it was concluded that an additional RT-PCR HRM assay with primer pair LR3.HRM6 is necessary. Primer pair LR3.HRM6 could differentiate between variants of groups I and II based on the duplex artificial mixed infection analysis (Figure 4G).

### Real-time RT-PCR and HRM analysis

One hundred and sixty nine grapevine samples were screened with the LR3.HRM4 primer pair of which 48 samples tested negative for GLRaV-3. From the remaining 121 samples positive for GLRaV-3, 35 were positive for group III variants and 87 positive for group VI variants. One hundred and two samples were positive for group I and/or group II variants. These 102 samples were screened with the LR3.HRM6 primer pair to determine their variant status and 14 samples were found to be infected with group I variants and 88 samples infected with group II variants. Of the 121 GLRaV-3 positive samples, 73 samples had multiple infections (Table 3).

**Table 3:** Analysis of grapevine leafroll-associated virus 3 (GLRaV-3) single and mixed variant group infections

		**Variant group**	**Number of infections**
Single infections	48	I	7
		II	17
		III	0
		VI	24
Mixed infections	73	I + II	0
		I + III	0
		I + VI	1
		II + III	10
		II + VI	33
		III + VI	1
		I + II + III	0
		I + II + VI	4
		II + III + VI	22
		I + II + III + VI	2
Total	121		

In order to use RT-PCR HRM with the LR3.HRM4 primer pair for variant differentiation, a confidence interval for each variant group’s melting point was determined. The sample melting points for groups II, III and VI were not normally distributed (Table 4), resulting in less than 95% of the melting points to fall within the interval ±1.96 standard deviations from the mean. All RT-PCR HRM reactions were performed in duplicate and yielded consistent melting points per sample, however, significant variation was observed between samples from the same variant group. This can probably be explained by the existence of quasispecies that arose from the high mutation rate of the viral genome [22,23]. An average melting point was therefore not adequate to differentiate GLRaV-3 variant groups. A melting point temperature interval was consequently calculated to include 95% of the melting points observed. The 2.5^th^ and 97.5^th^ percentiles for each variant group (Table 4) were used to calculate the limits of the interval to include 95% of the data. The largest possible interval for each variant group was also determined to include the highest number of melting points for each variant group without overlapping with the adjacent interval (Table 4). Intervals where all data points (100% confidence) fell within the maximum range, the limits were adjusted to the 2.5^th^ to 97.5^th^ percentile to incorporate a margin of error to ensure accurate classification. The confidence intervals of groups I and II overlapped almost completely and therefore differentiation was not possible. The group I and II intervals could not be separated and a joint interval from 83.22°C to 84.18°C (95% confidence) was calculated for samples from variant groups I and/or II. For groups III and VI the intervals were calculated as 84.57°C to 85.64°C (95.89% confidence) and 85.65°C to 86.37°C (92.96% confidence), respectively.

**Table 4:** **Calculation of the melting point confidence interval for each variant group based on real-time RT-PCR HRM curve analysis using LR3.HRM4 or LR3.HRM6 primer pairs**^**a**^

**Variant group**	**2.5**^**th**^**percentile**^**b**^	**97.5**^**th**^**percentile**^**c**^	**Interquartile range (IQR) (75%-25%)**^**d**^	**Number of outliers (>±1.5xIQR)**	**Shapiro- Wilk test of normality (p)**^**e**^	**Melting point interval without overlaps**		
								**Confidence (%)**
**LR3.HRM4**								
Group I	**83.22**	84.08	0.43	0.00	0.103^e^	83.20	83.70	67.74
Group II	83.22	**84.18**	0.45	0.00	0.000	83.15	84.56	100
Group III	84.91	85.65	0.13	6.00	0.000	**84.57**	**85.64**	95.89
Group VI	85.35	86.28	0.15	12.00	0.000	**85.65**	**86.37**	92.96
**LR3.HRM6**								
Group I	**84.79**	**85.39**	0.09	7.00	0.002	84.78	85.69	100
Group II	**86.01**	**86.78**	0.42	0.00	0.000	85.70	86.90	100

^a^The data generated for each variant group was tested for normality in order to calculate the largest interval with the highest confidence without overlaps between variant groups. These intervals are indicated in bold. Intervals where all data points (100% confidence) fell within the maximum range, the limits were adjusted to the 2.5^th^ to 97.5^th^ percentile to incorporate a margin of error to ensure accurate classification.

^b^2.5^th^ percentile is the melting point temperature where 2.5% of data points is less than or equal to that temperature.

^c^97.5^th^ percentile is the melting point temperature where 2.5% of data points is greater than or equal to that temperature.

^d^Interquartile range is the interval where the middle 50% of melting point temperatures can be expected.

^e^Assume a normal distribution if p > 0.05, meaning approximately 95% of melting point temperatures of the variant group will be within ±1.96 standard deviations of the mean.

To differentiate groups I and II, primer pair LR3.HRM6 was used. The melting points of both groups I and II were also not normally distributed and the confidence intervals were calculated using the 2.5^th^ to 97.5^th^ percentile range. The group I interval was calculated from 84.79°C to 85.39°C (95% confidence) and for group II from 86.01°C to 86.78°C (95% confidence).

Outliers were identified within variant groups III and VI for primer pair LR3.HRM4 and within variant group I for primer pair LR3.HRM6 (Table 4). The comparatively high number of outliers identified within variant group VI resulted in a lower confidence level for this variant group compared to the other groups.

The Rotor-Gene software can perform automated variant classification based on the melting point interval calculated from the derivative melting curve (dF/dT) profile for each sample. Bins were programmed based on the data set for each variant group that consisted of a calculated midpoint with a 95% confidence interval width. This allows the software to automatically classify each melting peak observed according to the bins programmed. To avoid unnecessary peak calling, the temperature threshold can be set at 83°C, because none of the variant groups is expected to have a melting point below 83°C.

These confidence intervals for both primer pairs LR3.HRM4 and LR3.HRM6 were calculated, based on data generated from RNA extracted using the CTAB method. It was observed that when a different RNA extraction protocol was used, the melting points for each variant group shifted proportionally (unpublished data). This is probably the result of the interaction of the intercalating SYTO 9 dye which is influenced by inhibitors and salt concentration in the RNA extract.

In this study, preliminary data on the incidence of GLRaV-3 variants in the Western Cape of South Africa were collected using RT-PCR HRM analysis. A previous study, using SSCP, identified variant group II as the most prevalent, with 54% of a sample size of 80 being infected by this variant [24]. In the present study, variant groups II and VI were equally distributed with a 39% infection rate each. Of the 224 infections detected in 121 positive samples, 21% were single variant infections, with half of these classified as group VI. These preliminary data is not necessarily an indication of the distribution of GLRaV-3, since more than halve of the grapevine samples came from only three severely infected vineyards and the rest were from greenhouse isolate collections that decreases the complexity of mix infections. However, it confirms the presence of four GLRaV-3 variant groups in South Africa and that the technique can successfully be applied to study the distribution of GLRaV-3 variants.

### Variant status confirmation using multiplex RT-PCR

**Figure 5:**
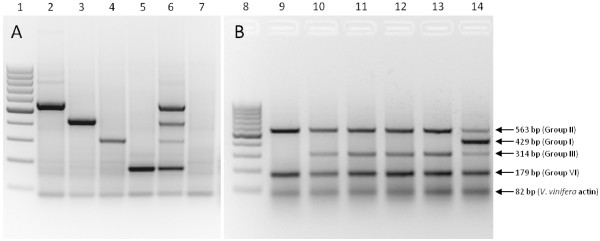
**Agarose gel electrophoresis of multiplex RT-PCR amplicons.** Visualization of multiplex RT-PCR amplicons separated on a 2% TAE agarose gel with ethidium bromide staining. **Figure**5**A** represents grapevine samples singly infected with one GLRaV-3 variant group and **Figure**5**B** represents field samples with multiple infections. Lane 1: Fermentas GeneRuler 100 bp DNA ladder, Lane 2: Group II variant (563 bp) and *V. vinifera* actin (82 bp), Lane 3: Group I variant (429 bp) and *V. vinifera* actin, Lane 4: Group III variant (314 bp) and *V. vinifera* actin, Lane 5: Group VI variant (179 bp) and *V. vinifera* actin, Lane 6: Group I, II, III and VI variants and *V. vinifera* actin, Lane 7: Negative control, Lane 8: Fermentas GeneRuler 100 bp DNA ladder, Lane 9: Group II, VI and *V. vinifera* actin, Lane 10: Group II, III, VI and *V. vinifera* actin, Lane 11: Group II, III, VI and *V. vinifera* actin, Lane 12: Group II, III, VI and *V. vinifera* actin, Lane 13: Group II, III, VI and *V. vinifera* actin , Lane 14: Group I, II, III, VI variants and *V. vinifera* actin.

The multiplex RT-PCR was optimized to detect GLRaV-3 variant groups I, II, III and VI in a single reaction (Figure 5). Two reverse primers targeting GLRaV-3 ORF1a and the *V. vinifera* actin gene, respectively, were used for the cDNA synthesis. The PCR was optimized to produce a single amplicon for each variant group and the internal control. The reaction was tested with and without the addition of bovine serum albumin (BSA), but without BSA the amplification was sub-optimal for GLRaV-3 variant groups I and II. The addition of BSA has previously been shown to enhance the amplification efficiency of targeted DNA by stabilizing enzymes and neutralizing inhibitory contaminants [25-27]. One hundred and twenty one GLRaV-3 positive samples were screened using the multiplex RT-PCR protocol. Thirteen of these samples were positive for group I variants, 87 samples positive for group II variants, 32 samples positive for group III samples and 80 samples positive for group VI variants. The multiplex RT-PCR validated 94% of the infections detected by the combined LR3.HRM4 and LR3.HRM6 RT-PCR HRM assays, indicating that the RT-PCR HRM is more sensitive than the multiplex RT-PCR. This is not unexpected, because of the specificity of the instrument used and the primer target region selected. The multiplex RT-PCR was designed to target the 5’UTR of the GLRaV-3 genome due to the high variability in this region. Insertions and deletions in this region made it an ideal target for the design of variant-specific primers. The 5’UTR is only represented in the genomic RNA whereas the Hsp70h is also represented in sub-genomic RNAs produced during GLRaV-3 replication. This implies an increased number of templates for the 3’ half of the genome [3,28], making the Hsp70h region a better-suited target for viral diagnostics by improving sensitivity. Another advantage of the RT-PCR HRM is that it will be possible to identify a new variant group if a distinct melting curve profile is produced. With the multiplex RT-PCR a new variant group will remain undetected or unidentified if the primers are also specific for the new variant.

## Conclusion

In order to investigate the spread and impact of different GLRaV-3 variants in vineyards, sensitive diagnostic techniques are a necessity. Serological tests like ELISA is one of the preferred detection methods for plant viral disease diagnostics due to its simplicity and effectiveness [29]. However, as viral sequences become available, virus-specific primers can be designed to be used in RT-PCR or real-time RT-PCR that is more sensitive than serological tests. In this study, a real-time RT-PCR was designed that is able to detect GLRaV-3 variant groups I, II, III and VI, using a single primer pair targeting the Hsp70h gene of GLRaV-3. If HRM curve analysis is added to the real-time RT-PCR, it is possible to differentiate between variant groups based on three melting point intervals. An additional primer pair was identified that is able to differentiate between variant groups I and II. The RT-PCR HRM assay provides a more sensitive, automated and rapid tool to detect and differentiate between different GLRaV-3 variant groups. The multiplex RT-PCR offers an end-point PCR alternative to differentiate between the variant groups present in South African or to be used as a validation method for the RT-PCR HRM. The abovementioned tools will contribute to the understanding of the pathogenesis of leafroll disease and aid epidemiology studies to investigate how these different GLRaV-3 variant groups are spreading.

## Materials and methods

### Virus source and sample preparation

Plant material from 173 grapevine plants was used to establish and validate the RT-PCR HRM. Forty vines from a study in 2008, where the distribution of GLRaV-3 variants in disease clusters were investigated, were re-collected from a vineyard in the Worcester vine growing region [24]. Ninety grapevine plants were randomly selected during a field survey in 2008 from two severely infected vineyards in the Stellenbosch area and 39 grapevine samples were from a virus isolate collection (Vitis Laboratory, Stellenbosch University, South Africa), maintained in *V. vinifera*, grown in the greenhouse. An additional GLRaV-3 positive sample for each variant group, singly infected with only that variant (Group I, II, III and VI), were obtained from a virus isolate collection (ARC-Plant Protection Research Institute, Pretoria, South Africa). Phloem scrapings were prepared from cane material collected during winter. Total RNA was extracted from 2.5g phloem tissue using an adapted Cetyltrimethylammonium bromide (CTAB) method (2% CTAB, 2.5% PVP-40, 100mM Tris-HCL pH8, 2M NaCl, 25mM EDTA pH8 and 3% β-mercaptoethanol) [30].

### Primer design

Conserved regions in the GLRaV-3 genome were used to design primer pairs that are able to detect the four GLRaV-3 variant groups found in South Africa. These conserved regions had to be in close proximity to result in amplicons with lengths of 150-300 base pairs (bp). Representative isolates of GLRaV-3 variant groups with complete genome sequences available [GenBank: GQ352631.1, GenBank: EU259806.1, GenBank: GQ352632.1, GenBank: GQ352633.1, GenBank: JQ655295, GenBank: GU983863.1, GenBank: AF037268.2, GenBank: EU344893.1] were used to identify the conserved regions by constructing a multiple sequence alignment using BioEdit 7.0.5.3 [31]. The partial isolate NZ-1 sequence was also included in the multiple sequence alignment [GenBank: EF508151.1]. Six primers pairs were identified targeting ORF1a, ORF1b, ORF4 and ORF6 (Table 5). The six primers pairs were tested on samples singly infected with a specific GLRaV-3 variant group using the real-time RT-PCR to identify which primer can most effectively detect all variants and possibly differentiate between them by using HRM curve analysis.

**Table 5:** List of primers used with the real-time RT-PCR HRM assay and the end-point multiplex RT-PCR protocol

**Primer pair**	**Sequence (5'-3')**	**Target region**	**Amplicon size (bp)**
LR3.HRM1.F	TAGACGTTAAAGATGTGAAGCG	GLRaV-3 ORF1a	167
LR3.HRM1.R	TCGTACACATCCACCATA		
LR3.HRM2.F	GTCCTAGATTCGGATTTTGTCG	GLRaV-3 ORF1a	231
LR3.HRM2.R	GAATACTCTTCGCCCTATC		
LR3.HRM3.F	CTGGTTGCTTTCGAGGTATATGAG	GLRaV-3 ORF1b	295
LR3.HRM3.R	CACTTCAAGGTGTTGCGCTT		
LR3.HRM4.F	TAATCGGAGGTTTAGGTTCC	GLRaV-3 ORF4	226
LR3.HRM4.R	GTCGGTTCGTTAACAACAC		
LR3.HRM5.F	TGTGTAAGAAGGTTATGGG	GLRaV-3 ORF6	224
LR3.HRM5.R	TACTGCCTTACCGGGTTTTC		
LR3.HRM6.F	GTCACCAGGTGTTCCAAACC	GLRaV-3 ORF1a	305
LR3.HRM6.R	AACGCCCTGTATGTCCTCTC		
LR3_Universal_F	TAAATGCTCTAGTAGGATTC	GLRaV-3 5'UTR	
621_430R	TAACCCAACACGACGATGAG	GLRaV-3 5'UTR	429^a^
623_564R	CTCACGCTAAACACACCAAG	GLRaV-3 5'UTR	563^a^
PL20_315R	GTTTGTAACAAAGAAACACG	GLRaV-3 5'UTR	314^a^
GH11_180R	CCAAAACGAAGACGAAAAGAAGAG	GLRaV-3 5'UTR	179^a^
LR_ORF1aR	CGTCCGCTTCACCCCTTTGG	GLRaV-3 ORF1a	
Vv_Actin_F [32]	CTTGCATCCCTCAGCACCTT	*V. vinifera* predicted actin-7	82
Vv_Actin_R [32]	TCCTGTGGACAATGGATGGA		

^a^Amplicon size if use together with LR_Universal_F.

### Verification of one-step real-time RT-PCR assay with melting curves generated from plasmid DNA

Real-time RT-PCR amplicons of GLRaV-3 variant groups I, II, III and VI were cloned into a pGEM-T-easy Vector (Promega) and sequenced to obtain variant-specific plasmid DNA. Artificial *in vitro* mixed infections between the variant-specific plasmid DNA were made to determine whether the chosen primer pair could differentiate between variants if mixed infections would be present in field plants. Duplex infections were made in a 1:3, 1:1 and 3:1 ratio for each combination of two variant groups. Reaction mixtures of all variant-specific plasmid DNA PCR HRM assays contained 1x KAPA Taq Buffer A (KAPA Biosystems), 0.4 μM reverse primer (IDT), 0.4 μM forward primer (IDT), 0.2 mM dNTP mix (Fermentas) 1 μM SYTO 9 (Invitrogen), 0.04 U/ μl KAPA Taq DNA polymerase (KAPA Biosystems) and 0.01 ng/μl plasmid DNA. Cycle conditions included an initial denaturation step at 94°C for 5 minutes, followed by 45 cycles of 94°C for 10 seconds, annealing at 55°C for 10 seconds and elongation at 72°C for 20 seconds. Acquisition on the green channel was recorded at the end of the extension step. High-resolution melting curves of PCR amplicons were obtained with temperatures ranging from 70°C to 90°C with a 0.1°C increase in temperature every two seconds.

### Real-time RT-PCR and HRM analysis

The primer pair that could most effectively detect and differentiate between GLRaV-3 variant groups I, II, III and VI was used to screen the 173 samples to optimize the assay. Each reaction was performed in duplicate using the RT-PCR HRM on a Qiagen Rotor-Gene Q thermal cycler. Reaction mixtures contained 1x KAPA Taq Buffer A (KAPA Biosystems), 0.4 μM reverse primer (IDT), 0.4 μM forward primer (IDT), 0.2 mM dNTP mix (Fermentas), 1 μM SYTO 9 (Invitrogen), 0.04 U/μl KAPA Taq (KAPA Biosystems), 0.08 U/μl Avian Myeloblastosis Virus (AMV) reverse transcriptase (Fermentas) and 100 ng RNA. Optimized cycle conditions were a cDNA synthesis step at 48°C for 30 minutes, an initial denaturation step at 94°C for 5 minutes, followed by 45 cycles of 94°C for 10 seconds, annealing at 55°C for 10 seconds and elongation at 72°C for 20 seconds. Acquisition on the green channel was recorded at the end of the extension step. High-resolution melting curves of PCR amplicons were obtained with temperatures ranging from 70°C to 90°C with a 0.1°C increase in temperature every two seconds. HRM curve analysis was performed using the Rotor-Gene software version 1.7. In order to use the RT-PCR HRM to differentiate between variants, a melting point confidence interval had to be determined for each variant group. The data generated for each variant group were tested for normality using the Shapiro-Wilk algorithm and descriptive statistics were calculated using the SPSS statistics software package 19 (IBM).

### Variant status conformation using multiplex RT-PCR

Variant-specific RT-PCR reverse primers (Table 5) targeting the 5’ UTR of the GLRaV-3 variant groups I, II, III and VI were designed to be used in a single reaction with one forward primer. This multiplex RT-PCR was designed to validate the HRM analysis and assign each sample to a specific variant group. A primer pair targeting the *V. vinifera* actin gene was also included in the multiplex RT-PCR to act as an RNA specific internal control. A two-step RT-PCR multiplex protocol was used and approximately 1000-1500 ng of total RNA was denatured at 65°C for 5 minutes with 2 μM of LR_ORF1aR primer (IDT) and 2 μM of Vv_Actin_R (IDT) [32] (Table 5) and incubated for 2 minutes on ice (5 μl final volume). The RNA was reverse-transcribed by incubation at 48°C for 1 h in a reaction mixture (10 μl final volume) containing 1x Avian Myeloblastosis Virus (AMV) reverse transcriptase buffer (Fermentas), 1 mM dNTP mix (Fermentas), 1U/μl Ribolock (Fermentas) and 0.5 U/μl AMV reverse transcriptase (Fermentas). A 2.5 μl aliquot of cDNA was subjected to PCR in a 25 μl reaction mixture containing 1x KAPA Taq buffer B (KAPA Biosystems), 0.4 mM dNTP mix (Fermentas), 0.4 μM LR_universal_F primer (IDT), 0.28 μM Vv_Actin F (IDT) [32], 0.28 μM Vv_Actin R (IDT) [32], 0.4 μM of each variant-specific reverse primer (IDT) (Table 5), 0.5μg/μl Bovine Serum Albumin (BSA) (Roche) and 0.08 U/μl KAPA Taq DNA polymerase (KAPA Biosystems). Cycle conditions included an initial denaturation step at 94°C for 5 minutes, followed by 35 cycles of 94°C for 30 seconds, annealing at 58°C for 20 seconds and elongation at 72°C for 40 seconds. Final extension was at 72°C for 7 minutes. Amplicons were visualized on an ethidium bromide-stained 2% TAE-agarose gel (2 M Tris, 1M glacial acetic acid, 0.05 M Na_2_EDTA, pH 8).

## Competing interest

The authors declare that they have no competing interests.

## Authors’ contributions

RB contributed to the design of the study, primer design for the RT-PCR HRM assay, sample collection, RNA extractions, optimization of the RT-PCR HRM assay, screening of samples, statistical analysis, designing the multiplex RT-PCR, validation of RT-PCR HRM assay with multiplex RT-PCR and drafting the manuscript. AECJ designed primers for the RT-PCR HRM assay, supplied single variant infected GLRaV-3 source plants, contributed to the design of the study, sample collection, RNA extractions, optimization of RT-PCR HRM assay and drafting of the manuscript. HJM contributed to the design of the study, primer design, sample collection, statistical analysis, designing the multiplex RT-PCR protocol and drafting of the manuscript. JTB contributed to the design of the study, data analysis and drafting the manuscript. All authors read and approved the final manuscript.
